# The use of the cluster randomized crossover design in clinical trials: protocol for a systematic review

**DOI:** 10.1186/2046-4053-3-86

**Published:** 2014-08-12

**Authors:** Sarah J Arnup, Andrew B Forbes, Brennan C Kahan, Katy E Morgan, Steve McDonald, Joanne E McKenzie

**Affiliations:** 1 School of Public Health and Preventive Medicine, Monash University, Level 6, The Alfred Centre, Melbourne, VIC 3004, Australia; 2 Pragmatic Clinical Trials Unit, Queen Mary University of London, 58 Turner St, London E1 2AB, UK

**Keywords:** Cluster randomized trial, Crossover, Intra-cluster correlation, Sample size, Design, Statistical analysis, Reporting

## Abstract

**Background:**

The cluster randomized crossover (CRXO) design is gaining popularity in trial settings where individual randomization or parallel group cluster randomization is not feasible or practical. In a CRXO trial, not only are clusters of individuals rather than individuals themselves randomized to trial arms, but also each cluster participates in each arm of the trial at least once in separate periods of time.

We will review publications of clinical trials undertaken in humans that have used the CRXO design. The aim of this systematic review is to summarize, as reported: the motivations for using the CRXO design, the values of the CRXO design parameters, the justification and methodology for the sample size calculations and analyses, and the quality of reporting the CRXO design aspects.

**Methods/Design:**

We will identify reports of CRXO trials by systematically searching MEDLINE, PubMed, Cochrane Methodology Register, EMBASE, and CINAHL Plus. In addition, we will search for methodological articles that describe the CRXO design and conduct citation searches to identify any further CRXO trials. The references of all eligible trials will also be searched.

We will screen the identified abstracts, and retrieve and assess for inclusion the full text for any potentially relevant articles. Data will be extracted from the full text independently by two reviewers. Descriptive summary statistics will be presented for the extracted data.

**Discussion:**

This systematic review will inform both researchers addressing CRXO methodology and trialists considering implementing the design. The results will allow focused methodological research of the CRXO design, provide practical examples for researchers of how CRXO trials have been conducted, including any shortcomings, and highlight areas where reporting and conduct may be improved.

## Background

The most commonly used experimental design to assess the effects of an intervention is the individually randomized parallel two-arm trial [1]. However, randomizing individuals is not always possible, and in many circumstances groups of people, or ‘clusters’, are instead randomly allocated to the intervention groups. Cluster randomization is commonly used in the following situations: when contamination may occur if individuals in the same cluster were randomized to different intervention groups, the intervention is targeted at the cluster level, or for logistical, feasibility, or ethical reasons [2].

Individuals within a cluster tend to have more similar outcomes than individuals across clusters. For example, due to case-mix differences of patients presenting to different hospitals, patients in the same hospital may have more similar outcomes than patients across different hospitals. As a result, a cluster randomized trial usually requires a larger sample size than an individually randomized trial in order to achieve the same power to detect the same difference between groups. Failure to account for the clustering during analysis can lead to overly precise estimates of the intervention effect and hence potentially incorrect inferences about the effectiveness of the intervention [2].

A variation of the parallel group cluster randomized design is the cluster randomized crossover design (CRXO). In the CRXO design each cluster receives each intervention at least once in separate periods of time [3,4]. During each time period the cluster may contain different individuals, the same individuals, or a mixture of both different and same individuals [5].

Analogous to trials where individuals are randomized and a crossover is included in the design to improve efficiency, incorporating a crossover into a parallel group cluster randomized design increases efficiency if the cluster environment remains similar between time periods [5]. The gains in efficiency of a CRXO trial over a parallel group cluster randomized trial depend upon the number of clusters, the size of the clusters, the number of time periods, and the similarity between individuals within the trial. The similarity in the outcomes of individuals within a cluster within a time period is typically measured by the within-cluster within-period intra-cluster correlation coefficient (ICC). The similarity between individuals within the same cluster, both within the same time period and across different time periods, is typically measured by the within-cluster between-period ICC [4,6].

To our knowledge, there have only been limited reviews of the CRXO trial design. These reviews have taken place in the introductory sections of methodological papers with the purpose of illustrating the design and highlighting the need for appropriate methods of analysis [3,4,6,7]. Turner *et al*. [3] reviewed eight trials [8-15] from 1985 to 2003 and noted that the majority of these trials did not allow for the within-cluster within-period and within-cluster between-period correlations in the analysis of outcomes. In the one trial [8] that did allow for these correlations in the analysis by using hierarchical modelling, Turner *et al*. [3] noted that no justification was given for the choice of analysis.

The CRXO design is gaining popularity in settings where cluster randomization is required, but the parallel group cluster randomized design is not practical because it leads to a prohibitively large sample size. However, no systematic review of the use of the CRXO design has been performed to date. Such a review will inform both researchers addressing CRXO methodology and trialists considering implementing this design.

### Objectives

The purpose of this systematic review is to establish from CRXO publications: the motivations for using the CRXO design, the values of the CRXO design parameters, the justification and methodology for the sample size calculations and analyses, and the quality of reporting the CRXO design aspects.

## Methods/Design

### Search methods for identification of studies

We will search for reports of CRXO trials that were conducted in humans and reported in English up until April 2014. One author (SA) will search for articles indexed in MEDLINE, PubMed, Cochrane Methodology Register, EMBASE and CINAHL Plus. (The search strategies for Ovid MEDLINE and the additional databases are in Appendix 1). Ovid was chosen to search MEDLINE because proximity searches, which cannot be performed in PubMed, are an essential component of the search strategy. As PubMed contains some additional publications not found in MEDLINE, a modified but less sensitive search will be performed using PubMed.

To supplement the above searches, SA will search CRXO methodology articles for further references to CRXO trials. A citation search of all identified methodology articles will be performed in Web of Science. SA and JM will identify CRXO methodology articles from PubMed using the following search strategy: ((cluster[tiab] AND cross*over[tiab]) OR cluster-crossover[tiab]) AND (method*[tiab] OR design[tiab] OR calcul*[tiab] OR analy*[tiab]).

Finally the references of all eligible articles will be screened by SA for further CRXO trials. If the title of the article or the text of the referring article suggests a CRXO design was used in the trial then the full text will be screened for eligibility by two reviewers (SA and AF or JM). This process will continue until no further eligible articles are identified.

### Inclusion criteria

We will include reports of CRXO trials with the following elements: the trial was undertaken in humans; the allocation of the intervention was to clusters of individuals rather than individuals themselves - the allocation does not have to be at random, since the statistical considerations remain the same irrespective of the method used to allocate clusters to the sequence of interventions; and each cluster received each intervention in a sequence over time (conventional crossover design), or at least some clusters crossed over from one intervention to another (such as two-treatment-four-sequence designs AA, AB, BA, and BB).

### Study selection

Titles and abstracts of all articles identified through the electronic searches will be imported into EndNote (EndNote X6, Thomson Reuters, New York, USA) and duplicates removed. Each abstract and title will assessed by one of five reviewers and a further 50% of abstracts will be assessed independently by a second reviewer. Full text articles will be retrieved when both reviewers answer ‘yes’ or ‘unclear’ to all selection criteria. The full text will not be retrieved if both reviewers agree that at least one selection criteria was not met. The full text will be retrieved for the remaining articles where all selection criteria assessed as ‘no’ by one reviewer were assessed as ‘yes’ or ‘unclear’ by the other reviewer.

Two reviewers will assess the full text articles. Trials will be included in the review if both reviewers agree that all selection criteria are met. Trials will be excluded if both reviewers agree that at least one selection criteria was not met. For the remaining trials, the decision to include the trial in the review will be by consensus between the two reviewers or by referral to a third reviewer.

### Data extraction and management

Two reviewers will independently extract data using an electronic data extraction form developed for this review (see Additional file 1). The data extraction form has been piloted by five reviewers in one to three studies each and adjusted accordingly.

We will extract data for each trial on: identification of the design in the title or abstract, justification for using the design, acknowledgement of the underlying assumptions of the design, demographic details (country, setting, unit of clustering, type of intervention, and control), characteristics, methods used in the trial (recruitment, randomization, allocation, and blinding), reporting of baseline characteristics of the trial design, and statistical analysis (methods to estimate intervention effects and adjustment for covariates). The extracted design characteristics will include: number of clusters, number of periods, number of cluster-periods (clusters × periods), number of individuals in the trial, number of interventions and the allocation of interventions to cluster periods, the variability of the number of individuals between cluster-periods, the reported measure of similarity between the outcomes of individuals within a cluster within a given period, and the reported measure of similarity between outcomes of individuals within a cluster between different periods.

The extracted data will include verbatim free text and categorization of the text into pre-specified options where possible. Any free text that does not fall into the pre-specified options will be categorized through discussion between reviewers. If data are not reported in the article or are incomplete, ‘not stated’ will be recorded on the data extraction form. Trialists will not be contacted, since we intend to examine trialists’ reported motivations. Differences in data extraction will be resolved through discussion until consensus is reached, or by referral to a third reviewer.

### Analysis

The flow of information through the systematic review will be reported in accordance with the PRISMA statement [16].

We will calculate descriptive summary statistics using frequencies and percentages of responses to categorical data. Free text will be classified and frequencies and percentages of the categories will be presented in the analysis. For continuous data the range and mean with SD or median with IQR will be presented as appropriate.

## Discussion

Our systematic review is designed to establish the motivations for using the CRXO design, the values of the CRXO design parameters, how both the sample size calculations and analyses account for the correlation structure and the incorporation of any covariates, and the quality of reporting the CRXO design, including the reporting of the correlation structure.

### Strengths and limitations of our protocol

To our knowledge, this will be the first systematic review of CRXO trials with a rigorous and pre-specified methodology. We have pre-defined our screening and data extraction forms. Where possible, reviewers will classify article text according to pre-defined categories rather than categorize the free text after all the data has been captured. Pre-specifying the methodology and data collection reduces the risk of introducing bias into the review. The full text screening and data extraction will be performed by two reviewers. A subset of the abstract screening will be performed by two reviewers. The data abstraction form has been piloted on several articles by more than one reviewer.

A limitation of this review is the difficulty in identifying CRXO trials. Trials that use cluster randomization frequently do not use the word ‘cluster’ in the title or abstract, and it is often not apparent that the allocation of the intervention was at the cluster level unless the methods are read in the full text article [17]. In an attempt to limit missed studies, the search strategy encompasses units that are typically cluster randomized (such as schools or hospitals) and the references of all eligible articles will be searched. In addition, a search for CRXO methodology articles, and articles which cite them, will be undertaken to identify further trials.

CRXO designs may be employed in areas outside of clinical trials undertaken in humans, for example, variants of split-plot designs in agricultural sciences. There may be studies in behavioral, social, or educational sciences which will be missed by the search methodology employed in this review as our search is restricted to a limited number of databases. However, while the application of the CRXO design in these fields may be interesting from a methodological perspective, the focus of this systematic review is cluster randomization and crossover of interventions in human clinical trials in health.

We are interested in the design, methods, and motivations for using the CRXO design. Our ability to assess some of these elements may be limited because of missing and incomplete reporting in the trial publications. While contact with trial authors may help establish some of missing elements, we do not plan to contact authors since we wish to reflect the information as reported. Decision-makers are generally reliant on only the information within publications, and therefore examining the quality and completeness of reporting is important. Knowledge of the adequacy of reporting is an essential step in developing reporting guidelines for such trials, if a need is found [18].

Both the stepped wedge design and the split-cluster design have similarities with the CRXO design. However, these designs were not considered in this review. A systematic review of the stepped wedge design was performed by Mdege *et al*. [19]. The split-cluster design does not have distinct time periods, so any similarity between the sub-clusters at a single point in time is likely to be different in nature to the similarity in clusters between time periods.

### Implications of this research

Results from our systematic review will allow for focused methodological research of the CRXO design. The results will also provide practical examples for researchers of how CRXO trials have been conducted, including any shortcomings, and highlight areas where reporting and conduct may be improved.

## Appendix 1: Search strategies

### Ovid MEDLINE search

CROSS OVER TERMS

1. (cross-over or cross?over or "cross* over").tw.

2. (switch-over or switch?over or "switch* over" or switch-back or switch?back or "switch* back" or switched).tw.

3. ((change-over or change?over or "change* over") not ((change-over or change?over or "change* over") adj1 time)).tw.

4. (ab*ba* adj3 design*).tw.

5. exp Cross-Over Studies/

6. 1 or 2 or 3 or 4 or 5

CLUSTER ALLOCATION TERMS

7. ((unit$1 or school$1 or hospital$1 or cluster* or region$1 or ward* or practice* or communit* or population* or facility or facilities or practitioner*) adj15 random*).tw.

8. ((unit$1 or school$1 or hospital$1 or cluster* or region$1 or ward* or practice* or communit* or population* or facility or facilities or practitioner*) adj15 interven*).tw.

9. ((group* adj random*) or (group* adj interven*)).tw.

10. 7 or 8 or 9

HUMANS ONLY

11. Humans/

12. Animals/

13. 12 not 11

COMBINE CONCEPTS

14. 6 and 10

15. 14 not 13

### PubMed search

CROSS OVER TERMS

1. "cross-over"[tiab] OR crossover[tiab] OR "cross over" [tiab] OR "crossed over"[tiab]

2. "switch-over"[tiab] OR switchover[tiab] OR "switch over"[tiab] OR "switch-back"[tiab] OR switchback[tiab] OR "switch back"[tiab] OR switched[tiab]

3. (change-over[tiab] OR changeover[tiab] OR "change over"[tiab] OR "changed over"[tiab] OR "changes over"[tiab]) not ("change-over time"[tiab] OR "changeover time"[tiab] OR "change over time"[tiab] OR "changed over time"[tiab] OR "changes over time"[tiab])

4. ab*ba[tiab]

5. Cross-Over Studies[mh]

6. #1 OR #2 OR #3 OR #4 OR #5

CLUSTER ALLOCATION TERMS

7. (cluster-randomi*[tiab] OR "cluster randomized"[tiab] OR "cluster randomised"[tiab] OR "cluster randomization"[tiab] OR "cluster randomization"[tiab])

HUMANS ONLY

8. (Animals[mh] NOT Humans[mh])

COMBINE CONCEPTS

9. #6 AND #7

10. #9 NOT 8

11. #10 NOT MEDLINE[sb]

### EMBASE search via embase.com

CROSS OVER TERMS

1. (cross-over or crossover or "cross over" or "crosses over" or "crossed over" or "crossing over"):ti:ab

2. (switch-over or switchover or "switch over" or "switches over" or "switched over" or switch-back or "switchback" or "switch back" or "switches back" or "switched back" or switched):ti:ab

3. ((change-over or changeover or "change over" or "changes over" or "changed over") not ((change-over or changeover or "change over" or "changes over" or "changed over") near/1 time)):ti:ab

4. (abba near/3 design):ti:ab or (abba near/3 designs):ti:ab

5. “crossover procedure”/exp

6. #1 or #2 or #3 or #4 or #5

CLUSTER ALLOCATION TERMS

7. ((unit or units or school or schools or hospital or hospitals or cluster or clusters or region or regions or ward or wards or practice or practices or community or communities or population or populations or facility or facilities or practitioner or practitioners) near/15 (random or randomly or randomise or randomize or randomised or randomized or randomises or randomizes or randomisation or randomization)):ti:ab

8. ((unit or units or school or schools or hospital or hospitals or cluster or clusters or region or regions or ward or wards or practice or practices or community or communities or population or populations or facility or facilities or practitioner or practitioners) near/15 (intervene or intervention or interventions)):ti:ab

9. ((group or groups or grouped) near/1 (random or randomly or randomise or randomize or randomised or randomized or randomises or randomizes or randomisation or randomization)):ti:ab or ((group or groups or grouped) near/1 (intervene or intervention or interventions)):ti:ab

10. #7 or #8 or #9

HUMANS ONLY

11. ‘animal’ not ‘human’

COMBINE CONCEPTS

12. #6 and #10

13. #12 not #11

14. #13 not ‘medline’

### CINAHL Plus search

CROSS OVER TERMS

1. TI (("cross-over" or "cross?over" or "cross* over")) OR AB (("cross-over" or "cross?over" or "cross* over"))

2. TI (("switch-over" or "switch?over" or "switch* over" or "switch-back" or "switch?back" or "switch* back" or switched)) OR AB (("switch-over" or "switch?over" or "switch* over" or "switch-back" or "switch?back" or "switch* back" or switched))

3. TI ((("change-over" or "change?over" or "change* over") not (("change-over" or "change?over" or "change* over") n1 time))) OR AB ((("change-over" or "change?over" or "change* over") not (("change-over" or "change?over" or "change* over") n1 time)))

4. TI (ab*ba* n3 design*) OR AB (ab*ba* n3 design*)

5. (MH "Crossover Design")

6. S1 or S2 or S3 or S4 or S5

CLUSTER ALLOCATION TERMS

7. TI (((unit or units or school or schools or hospital or hospitals or cluster or clusters or region or regions or ward or wards or practice or practices or community or communities or population or populations or facility or facilities or practitioner or practitioners) n15 random*)) OR AB (((unit or units or school or schools or hospital or hospitals or cluster or clusters or region or regions or ward or wards or practice or practices or community or communities or population or populations or facility or facilities or practitioner or practitioners) n15 random*))

8. TI (((unit or units or school or schools or hospital or hospitals or cluster or clusters or region or regions or ward or wards or practice or practices or community or communities or population or populations or facility or facilities or practitioner or practitioners) n15 interven*)) OR AB (((unit or units or school or schools or hospital or hospitals or cluster or clusters or region or regions or ward or wards or practice or practices or community or communities or population or populations or facility or facilities or practitioner or practitioners) n15 interven*))

9. TI (((group* n1 random*) or (group* n1 interven*))) OR AB (((group* n1 random*) or (group* n1 interven*)))

10. S7 or S8 or S9

HUMANS ONLY

11. (MH "Human")

12. (MH "Animals")

13. S12 not S11

COMBINE CONCEPTS

14. S6 and S10

15. S14 not S13

16. Exclude MEDLINE

## Abbreviations

CRXO: Cluster Randomized CrossOver; ICC: Intra-cluster Correlation Coefficient.

## Competing interests

The authors declare that they have no competing interests.

## Authors’ contributions

SA drafted the manuscript, search strategies and data extraction form, and participated in the testing of the search strategy and data extraction form. AF conceived of the review, participated in the development and testing of the search strategy and data extraction form, and helped draft the manuscript. BK revised and tested the search strategy and data extraction form, and critically reviewed the manuscript. KM revised and tested the search strategy and data extraction form, and critically reviewed the manuscript. SM developed and tested the search strategy and critically reviewed the manuscript. JM participated in the development and testing of the search strategy and data extraction form, and helped draft the manuscript. All authors read and approved the final manuscript.

## Supplementary Material

Additional file 1 This additional file contains the data that will be extracted from included studies. Click here for file
