# Supplementary material for: The use of the cluster randomized crossover design in clinical trials: protocol for a systematic review
Source: Syst Rev. 2014 Aug 12;3:86. doi: 10.1186/2046-4053-3-86 (PMC4138528; doi:10.1186/2046-4053-3-86)
Supplement: Additional file 1 — This additional file contains the data that will be extracted from included studies. [file 2046-4053-3-86-S1.docx]

**CRXO systematic review data extraction form**

**Date: 15 May 2014**

The unit of analysis for the review is ***study or trial***, not article. In many cases a study will be split into multiple articles, i.e. a protocol or design article, an article reporting the primary outcome(s), and many other articles reporting secondary outcomes.

The primary outcome for the study will be defined from the following hierarchy:

- The first primary outcome in the protocol document or first published paper for the study if there is no protocol document.
- The outcome used for the sample size calculation.
- The first outcome listed in the methods section of the abstract.

**Section 1: Study Identifiers**

| Study ID | (Autocompleted) |
| --- | --- |
| Is the article a protocol paper? | 0=No, 1=Yes |
| First Author Surname | String |
| Publication Year | Integer, 1946 to 2014 |
| Journal | Categorical |
| Reviewer's initials | Categorical |
| Date of review | Date |
| Notes | Not to be analysed |

**Section 2: Full Text Screening**

Does the article report a research trial that used or planned to use a CRXO design which incorporated the following design elements (*The article will only be marked for inclusion if “yes” is answered to all):*

| Outcomes were measured on humans in a study or trial at either cluster or individual level. | (0=No, 1=Yes, 2=Unclear) |
| --- | --- |
| Allocation of the intervention was at cluster level (The allocation does not have to be at random). | (0=No, 1=Yes, 2=Unclear) |
| Each cluster received each intervention, or at least some clusters crossed over from one intervention to another (e.g. two-intervention-four-sequence designs AA, AB, BA, BB). | (0=No, 1=Yes, 2=Unclear) |
| Each cluster received each intervention in a sequence over time, rather than concurrently in time. | (0=No, 1=Yes, 2=Unclear) |

**Section 3: Title and Abstract**

**Rationale:** To assess how CRXO trials are identified in the title and abstract.

| **Title** |  |
| --- | --- |
| Is the trial identified as a **cluster** randomised **crossover** trial in title? (*note words ‘cluster’ and ‘crossover’ must be used, placement of hyphens is unimportant*) | (0=No, 1=Yes) |
| **Abstract** |  |
| Is the trial identified as a **cluster** randomised **crossover** trial in abstract? (*note words ‘cluster’ and ‘crossover’ must be used, placement of hyphens is unimportant*) | (0=No, 1=Yes) |
| If no, copy verbatim from abstract how the unit of randomisation was described in the abstract | TEXT |
| If no, copy verbatim from abstract how the cross over of interventions was described in the abstract | TEXT |

**Section 4: Justification for the CRXO design**

**Rationale:** To understand why researchers are using the CRXO design and how they justify that decision.

Why was the CRXO design chosen? *For each of the following points enter (0=Not Discussed, 1=Yes, 2=Unclear). Select as many points as apply.*

| *Justification given by authors for* ***cluster*** *randomisation* |  |
| --- | --- |
| Intervention can **only** act at the cluster level, and therefore impossible to randomise individually *(e.g. if the intervention is an educational program for health care practitioners, or a program implemented publicly via radio or newspaper, the intervention will reach a group of people)*. | (0=Not Discussed, 1=Yes, 2=Unclear) |
| Practical/ethical/cost/administrative difficulties with randomising at an individual level. | (0=Not Discussed, 1=Yes, 2=Unclear) |
| Contamination likely between participants at the level of person/people delivering the intervention *(e.g. an educational intervention may be delivered to health care practitioners, and it may impossible for them to only apply the intervention to some individuals in their care and not others. Therefore contamination would occur in an individually randomised trial).* | (0=Not Discussed, 1=Yes, 2=Unclear) |
| Contamination likely between participants in a cluster *(e.g. a behavioural intervention may be delivered to schools, and it may be impossible to prevent primary caregivers from exchanging experiences, thereby contaminating each arm of the trial in an individually randomised trial).* | (0=Not Discussed, 1=Yes, 2=Unclear) |
| To ensure intervention is fully delivered *(if it is expected that compliance with the trial protocol will be reduced if members of a cluster were individually randomised)* | (0=Not Discussed, 1=Yes, 2=Unclear) |
| Outcome data only available at cluster level | (0=Not Discussed, 1=Yes, 2=Unclear) |
| Other, specify | TEXT |
|  |  |
| *Justification given by authors for* ***crossover*** *design* |  |
| Increased efficiency to overcome loss of power through randomising in clusters (*i.e. the authors specifically cite a reduction in precision/power due to cluster randomisation or the ‘design effect’ as the reason for the crossover element).* | (0=Not Discussed, 1=Yes, 2=Unclear) |
| Reduced efficiency in estimating the intervention effect due to a limited number of clusters available for inclusion in the trial *(i.e. the authors cite the limited number of clusters as the reason for the crossover element).* | (0=Not Discussed, 1=Yes, 2=Unclear) |
| Clusters are expected to have very different characteristics from each other (*i.e. the authors cite that they expect or wish to allow for clusters being very different in characteristics which might affect the outcome, and wish to crossover so that each cluster ‘acts as own control’)* | (0=Not Discussed, 1=Yes, 2=Unclear) |
| Other, specify | TEXT |

**Section 5: Trial Objectives**

**Rationale:** What are the levels of the primary objective being addressed with the CRXO trial?

| Copy **verbatim** objective or hypothesis from Introduction | TEXT |
| --- | --- |
| Is the primary objective at the cluster level, individual level, or both | 1. Cluster level  2. Individual level  3. Both  4. Not stated  5. Not clear, explain |

**Section 6: Population Details**

**Rationale:** What settings are CRXO trial being used in?

| Disease or domain under study  (can select multiple) | 1. Cancer  2. Cardiovascular  3. Central nervous system/musculoskeletal  4. Digestive/endocrine  5. Nutritional and metabolic  6. Gynaecology  7. Pregnancy and birth  8. Infectious diseases  9. Mental health and behavioural conditions  10. Pathological conditions  11. Symptoms and signs  12. Respiratory disease  13. Urogenital  14. Blood and immune system  15. Ear and nose  16. Eye  17. General health  18. Genetic disorders  19. Injuries  20. Accidents and wounds  21. Mouth and dental  22. Skin  23. Other |
| --- | --- |
| Country of trial (List all if 5 or less, otherwise state multinational) | TEXT |
| Setting (select one) | 1. Primary care practices/health care clinics  2. Communities/geographical areas  3. Households/families  4. Aged care facility  5. Hospital  6. Schools  7. Workplaces  8. Other,specify |
| Do the methods define the cluster unit? | (0=No, 1=Yes, 2=Description unclear) |
| Clusters receiving intervention (select one) | 1. Primary care practices (practice includes multiple health care professionals) 2. Individual health professional 3. Communities/Residential areas 4. Households/families 5. Hospital, specify unit/ward type 6. Nursing home/aged care 7. Schools 8. Worksites 9. Other, specify |
| Additional comments about clusters receiving intervention | TEXT |

**Section 7: Study Design**

**Rationale:** This section is intended to capture both the key design features of the published CRXO trial and how the design features are reported.

*An answer of “yes” means that the design aspect could be reconstructed from the information provided in the article.*

| The number of participating clusters | Integer, 99 = Not reported |
| --- | --- |
| The number of periods | Integer, 99 = Not reported |
| The number of interventions |  |
| Intervention treatments (*active interventions*) | Integer, 99 = Not reported |
| Control treatments (*e.g. no treatment, usual care. Enter 0 if all interventions are active*) | Integer, 99 = Not reported |
| List the different unique intervention sequences, (*i.e. AB, BA; or AA, BB, AB, BA*. *Copy* ***verbatim*** *from text*) | TEXT |
| Do the authors ***discuss*** how many interventions each ***participant*** will receive, i.e. if the participant can remain in cluster for longer than one period? | (0=Not Discussed, 1=Yes, 2=Unclear) |
| Is each period ***designed*** to include the same or different participants? (*i.e. are measurements repeated or not repeated on participants?)* | (0=Same participants, 1=Different participants, 2=Not Stated) |
| Are there any other relevant design features that may lead to additional correlation within the outcomes? E.g. hierarchical designs where there is clustering at different levels; wards within hospitals, GPs within general practices. (*copy* ***verbatim*** *from text*) | TEXT |

**Section 8: Carry over**

**Rationale:** To describe whether the risk of carry over being is acknowledged and managed.

| Do the authors ***discuss*** the possibility of carry over of intervention effects between periods? | (0=Not Discussed, 1=Yes, 2=Unclear*),*  Page and paragraph number |
| --- | --- |
| Do the methods ***detail*** how the risk of carry over effects will be minimised by the study design *(e.g. washout period, different subjects in each period)* | (0=No, 1=Yes-Sufficient to replicate, 2=Yes-Insufficient to replicate description, 3=NA-Carry over not possible)  Copy in text verbatim on the details. |

**Section 9: Blinding, bias and consent**

**Rationale:** To understand how randomisation or allocation of interventions was performed, the risk of bias in CRXO trials, and the adequacy of reporting.

*An answer of “yes” means that the design aspect could be reconstructed from the information provided in the article.*

| **Allocation sequence** |  |
| --- | --- |
| Was the allocation sequence randomly generated?  *(Where random is taken to mean: random number table, computer random number generator, coin tossing, shuffling cards or envelopes, throwing a dice, drawing of lots, minimisation)* | (0=No, 1=Yes-Sufficient to replicate, 2=Yes-Insufficient to replicate, 3=Unclear) |
| **Selection bias** |  |
| *Research team* |  |
| Do the people allocating the intervention sequence to the ***clusters*** know what the intervention sequence is? *(Allocation concealment)* | (0=No, 1=Yes, 2=Unclear) |
| Do the people recruiting/identifying ***participants*** know which intervention sequence has been assigned to the cluster? | (0=No, 1=Yes, 2=Unclear, 3=All participants recruited/identified before cluster randomisation) |
| Can the people recruiting/identifying ***participants*** influence which people are recruited/identified for inclusion in the study? | (0=No, 1=Yes, 2=Unclear)  Provide text to justify judgement – *e.g. participants are identified systematically from administrative data so identifier cannot influence inclusion.* |
|  |  |
| *Individual participants* |  |
| Who provides consent for the individual ***participant*** to ***receive intervention***? | 1. Individual. Consent is given by individual prior to intervention.  2. Cluster level. Individual participant does not give consent for intervention and cannot opt out of intervention. Consent is given by cluster spokesperson.  3. Opt out. Individual participant does not give consent for intervention. Intervention will be given unless participant opts out of intervention. Consent is given by cluster spokesperson.  4. Delayed consent. Consent is obtained from individual or their next of kin to continue intervention, but intervention is initiated without individual consent. Initial consent is given by cluster spokesperson.  5. Other  6. Unclear |
| If the individual participant provides consent does the ***participant*** have knowledge of the intervention they will receive ***prior*** to consenting? | (0=No, 1=Yes, 2=Unclear, 3=NA (if option 2)) |
| Is the intervention concealed to ***participants*** ***during*** the study? (*I.e. is the intervention blinded?*) | (0=No, 1=Yes, 2=Unclear) |
|  |  |
| **Consent for data collection** |  |
| Does consent for ***data collection*** occur at the cluster level, individual level, or is not required? | (0=Not stated, 1=cluster, 2=individual, 3=not required, 4=Unclear) |
|  |  |
| **Performance bias** |  |
| Was the intervention concealed at cluster level (*i.e. were health care professionals delivering intervention blind to the intervention*)? | (0=No, 1=Yes, 2=Unclear) |
|  |  |
| **Detection bias** |  |
| Were any patient, or individual level, reported outcomes collected (*e.g. pain, depression)?* | (0=No, 1=Yes, 2=Unclear) |
| If yes, list patient or individual level reported outcomes | TEXT |
| Were any subjective outcomes (e.g. clinician rated depression, condition specific mortality) collected by study personnel, clinicians, or outcome assessors (i.e. not reported by the individual level participant)? | (0=No, 1=Yes, 2=Unclear) |
| If yes, list which outcomes you considered subjective | TEXT |
| If yes, were the study personnel, clinicians, or outcome assessors who were assessing the subjective outcomes blind to the intervention assignment? | (0=No, 1=Yes, 2=Unclear) |

**Section 10: Intervention**

**Rationale:** To describe the type of interventions being used in CRXO

Type of experimental intervention (select all that apply. 0=No, 1=Yes, 2 =Unclear)

| Educational/quality improvement interventions targeted at health care professionals (e.g., distribution of educational materials, outreach visits, audit and feedback) | (0=No, 1=Yes, 2 =Unclear) |
| --- | --- |
| Quality improvement interventions targeted at the organisation of health care or health delivery service (e.g., financial, shifting of professional roles, multi-disciplinary teams, integration of services, changes in setting or equipment, home visits by nurses) | (0=No, 1=Yes, 2 =Unclear) |
| Participant health promotion or educational intervention (e.g., promotion of breastfeeding, smoking cessation intervention, decision aid, disease screening promotion) | (0=No, 1=Yes, 2 =Unclear) |
| Direct participant therapeutic intervention (e.g., **experimental** intervention includes drug/vaccine/vitamin supplement, insecticide spraying, surgery, testing of new clinical pathway – distinguish from **indirect** changes to patient therapies as a result of guideline adherence) | (0=No, 1=Yes, 2 =Unclear) |
| Other, specify | TEXT |
| Details of experimental intervention | TEXT |

| Control intervention (select one) | 1. Not reported 2. No active intervention, i.e. usual care 3. Minimal application for experimental intervention 4. Placebo intervention 5. Other active intervention 6. Other, specify |
| --- | --- |
| Details of control intervention | TEXT |

**Section 11: Sample size**

**Rationale:** To assess how sample size calculations are being performed and justified

***Correlation terminology:***

Indiviudual i, Cluster j, Period k

Within-cluster within-period correlation: Corr(y_ijk, y_i'jk)

Within-cluster between-period correlation: Corr(y_ijk, y_i'jk')

*For the questions which ask for a justification, these are yes/no questions, either a justification was provided or it was not. However the “unclear” option remains because circumstances may arise where it isn’t clear if the question applies.*

| Was a sample size/power calculation presented? | (0=No, 1=Yes-Sufficient to replicate, 2=Yes-Insufficient to be reproduced, 3=Unclear) |
| --- | --- |
| Which outcome was the sample size calculation based on? | TEXT |
| What was the scale of the outcome? | 1. Continuous  2. Binary  3. Categorical  4. Count  5. Time to event  6. Other, specify |
| Was there a justification for number of periods? | (0=No, 1=Yes, 2=Unclear)  Page and paragraph number |
| Was there a justification for number of clusters? | (0=No, 1=Yes, 2=Unclear)  Page and paragraph number |
| Was there a justification for number of participants per cluster? | (0=No, 1=Yes, 2=Unclear)  Page and paragraph number |
| Were equal (as opposed to unequal) cluster sizes assumed in the calculation? | (0=Unequal, 1=Equal, 2=Unclear)  Page and paragraph number |
| Was the ***within-cluster within-period*** clustering taken into consideration in the calculation? | (0=No, 1=Yes, 2=Unclear)  Page and paragraph number |
| Please provide additional details about the cluster level clustering if "within-cluster within-period" does not capture the type of clustering | TEXT |
| If yes, what was the scale of the value? | 1=correlation  2=variance components  3=design effect  4=other, specify |
| If yes, what was the value? | Float |
| If yes, what was the reference or source for the value for the ICC? (*e.g. pilot study, previous published research, best guess, unpublished research)* | TEXT |
| Was the within-cluster between-period clustering taken into consideration in the calculation? | (0=No, 1=Yes, 2=Unclear)  TEXT |
| Please provide additional details about the period level clustering if "within-cluster between-period" does not capture the type of clustering | TEXT |
| If yes, what was the scale of the value? | 1=correlation  2=variance  3=other, specify |
| If yes, what was the value? | Float |
| If yes, what was the reference/source of the value for the ICC? (*e.g. pilot study, previous published research, best guess, unpublished research)* | TEXT |
| If a reference or method was provided for the sample size calculation, provide the reference or details of the method *(copy* ***verbatim*** *from article)* | TEXT |
| Any additional comments? | TEXT |

**Section 12: Outcomes and Results**

**Rationale:** To describe the outcome measures being assessed with the CRXO design, and how they were being assessed.

For the primary outcome from the study, and the first secondary outcome that is reported in the abstract that is of a different data type to the primary outcome, answer the following:

| Specify outcome (*copy* ***verbatim*** *from text*) | TEXT |
| --- | --- |
| Classify how the outcome was identified from the study: | Primary outcome:  1. First primary outcome in the protocol document or published article  2. The outcome used for the sample size calculation  3. The first outcome listed in the article abstract  Secondary outcomes:  4. First outcome reported in abstract that is of a different data type to the primary outcome |
| What type of data is the outcome? (select one) | 1=Continuous  2=Binary  3=Categorical  4=Count  5=Time to event  6=Other, specify |
| How was the statistical analysis concerning the intervention effect performed for the outcome? (*copy* ***verbatim*** *from text*) | TEXT |
| Was a justification given for the choice of analysis? *(E.g. Was a justification given for why they chose a multilevel individual level approach rather than a cluster level approach)* | (0=No, 1=Yes, 2=Unclear) |
| What references were provided for the statistical analysis and/or justification of the analysis? | TEXT |
|  |  |
| Was the within-cluster within-period clustering accounted for in the analysis of the outcome? | (0=No, 1=Yes, 2=Unclear) |
| Please provide additional details about the cluster level clustering if "within-cluster within-period" does not capture the type of clustering | TEXT |
| If yes, what was the scale of the clustering measure? | 1=ICC  2=variance components  3=coefficient of variation  4=not reported  5=other, specify |
| If yes, what was the value of the clustering measure? | 99=Not reported |
| Was the within-cluster between-period clustering accounted for in the analysis of the outcome? | (0=No, 1=Yes, 2=Unclear) |
| Please provide additional details about the period level clustering if "within-cluster between-period" does not capture the type of clustering | TEXT |
| If yes, what was the scale of the clustering measure? | 1=ICC  2=variance components  3=coefficient of variation  4=not reported  5=other, specify |
| If yes, what was the value of the clustering measure? | 99=Not Reported |
| Were any other levels of clustering accounted for in the analysis? (*copy* ***verbatim*** *from text*) | TEXT |
| Was the intervention effect adjusted for any covariates? | (0=No, 1=Yes, 2=Unclear) |
| If yes, were the covariates individual or cluster level? | (1=Individual, 2=Cluster, 3=Both, 4=Unclear) |
| If yes, was the adjustment performed at individual or cluster level? | (1=Individual, 2=Cluster, 3=Both, 4=Unclear) |
| Provide details covariate adjustment *(copy* ***verbatim*** *from article)* | TEXT |

**Section 13: Baseline Characteristics (Table 1)**

**Rationale:** To describe how the baseline characteristics were summarised (Table 1).

In the following table, select all statements that apply.

| By intervention (i.e. separate summaries for the control and the intervention groups) *(NA if same participants are included in both interventions)* | (0=No, 1=Yes, 2=NA) |
| --- | --- |
| By period (i.e. separate summaries for each period for each intervention) *(NA if same participants are included in both periods)* | (0=No, 1=Yes, 2=NA) |
| By cluster (I.e. a separate summary for each cluster) | (0=No, 1=Yes) |
| Other, specify | TEXT |

What were the sizes of the analysed clusters? If the values are not reported directly, but can be calculated from the supplied data, then perform the calculation and enter that value. I.e. 834 participants from 18 clusters gives a mean cluster size of 46.

| What was the mean cluster size ***overall***? (99 = Not determinable) | Float |
| --- | --- |
| Was an indication provided for the variation in cluster size between clusters? | (0=No, 1=Yes) |
| *If yes – copy text* ***verbatim*** *from article* |  |
|  |  |
| What was the mean cluster size in each ***intervention***? (99 = Not determinable) |  |
| Intervention 1 (name:) | Float, |
| *Insert rows for each intervention* |  |
|  |  |
| What was the mean cluster size in each ***period***? (99 = Not determinable) |  |
| Period 1 (name:) | Float, |
| *Insert rows for each intervention* |  |
|  |  |
| What was the mean cluster size in each ***intervention and period*** (ie in each cluster period)? (99 = Not determinable) |  |
| Period 1, Intervention 1 | Float |
| *Insert rows for each period and intervention* |  |
| Were any other summary statistics of the cluster sizes provided in the article? *(Copy text* ***verbatim*** *from article) (e.g. coefficient of variation, harmonic mean)* | TEXT |
| Was an indication provided for the variation in cluster size over time? *(e.g. between periods)* | (0=No, 1=Yes) |
| *If yes – copy text* ***verbatim*** *from article* | TEXT |

**Section 14: Missing data**

**Rationale:** To summarise whether missing data is being reported in the CRXO trials, and how the data is being accounted for in analyses

| Was missing data discussed in the article? | (0=No, 1=Yes) |
| --- | --- |
| How was missing data reported? (*copy* ***verbatim*** *from text*) | TEXT |
| How did the authors account for missing data in the analysis? (*copy* ***verbatim*** *from text*) | TEXT |
